# Supplementary material for: Anticancer Effects of 6-Gingerol through Downregulating Iron Transport and PD-L1 Expression in Non-Small Cell Lung Cancer Cells
Source: Cells. 2023 Nov 15;12(22):2628. doi: 10.3390/cells12222628 (PMC10670414; doi:10.3390/cells12222628)
Supplement: Supplementary file 1 [file cells-12-02628-s001.zip › cells-2680640-supplementary.pdf]

Table S1. q-PCR primer sequences and annealing temperature.

Sl No	Gene	Annealing temperature (°C)	Sequence (5` - 3`)
1	*iNOS*	58	F: 5′- TGCTCAGCTCATCCGCTATG -3′<br>R: 5′- GATGTTCCATGGCCACCTCA -3′
2	*p21*	58	F: 5′- ATGAAATTCACCCCCTTTCC -3′<br>R: 5′- AGGTGAGGGGACTCCAAAGT -3′
3	*p27*	58	F: 5′- CCGGCTAACTCTGAGGACAC -3′<br>R: 5′- TTGCAGGTCGCTTCCTTATT -3′
4	*p53*	58	F: 5′- AGGCCTTGGAACTCAAGGAT -3′<br>R: 5′- TGAGTCAGGCCCTTCTGTCT -3′
5	*CDK4*	58	F: 5′- CCCGAAGTTCTTCTGCAGTC -3′<br>R: 5′- CTGGTCGGCTTCAGAGTTTC -3′
6	*Cyclin D1*	58	F: 5′- TGTTTGCAAGCAGGACTTTG -3′<br>R: 5′- TCATCCTGGCAATGTGAGAA -3′
7	*Cyclin E*	58	F: 5′- ATCCTCCAAAGTTGCACCAG -3′<br>R: 5′- AGGGGACTTAAACGCCACTT -3′
8	*Bcl2*	58	F: 5′- TGGGGTCATGTGTGTGGA -3′<br>R: 5′- CCCAGCCTCCGTTATCCT -3′
9	*Bax*	58	F: 5′- ATGCGTCCACCAAGAAGC -3′<br>R: 5′- CAGCTGCCACTCGGAAAA -3′
10	*Cyto c*	58	F: 5′- CCCAAGCACTTCTGGTGG -3′<br>R: 5′- ATCACGCCATTGCACTCC -3′
11	*Capase 9*	58	F: 5′- GGACATGCTGGCTTCGTT -3′<br>R: 5′- TGGGTGTTTCCGGTCTGA -3′
12	*GAPDH*	58	F: 5′- AAGGCCATCACCATCTTCCA -3′<br>R: 5′- ACGATGCCAAAGTGGTCATG -3′
13	*miRNA*	miR-34a	5′- TGGCAGTGTCTTAGCTGGTTGT -3′
		miR-200	5′- TAATACTGCCGGGTAATGATGGA -3′
		miR-U6	5′- CTCGCTTCGGCAGCACA -3′

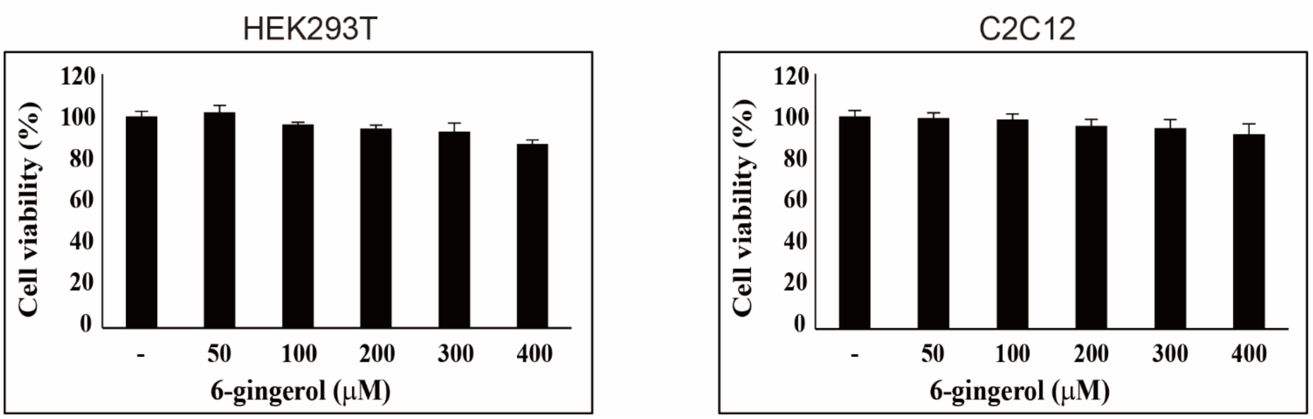

Figure S1. Cell viability assay by 6-gingerol on HEK 293 T and C2C12 cell viability.
